# Analysis of the clinical features of 980 accidental pediatric injuries in the PICU

**DOI:** 10.3389/fped.2025.1562237

**Published:** 2025-05-01

**Authors:** Yufan Yang, Wang Chen, Hengyun He, Xinping Zhang, Jiaotian Huang, Guanghui Zhu, Xiulan Lu, Zhenghui Xiao

**Affiliations:** ^1^ Department of Intensive Care Unit, The School of Pediatrics, Hengyang Medical School, University of South China (Hunan Children’s Hospital), Changsha, Hunan, China; ^2^ Department of Intensive Care Unit, The Affiliated Children’s Hospital of Xiangya School of Medicine, Central South University (Hunan Children’s Hospital), Changsha, Hunan, China; ^3^ Department of Orthopedics, The Affiliated Children’s Hospital of Xiangya School of Medicine, Central South University (Hunan Children’s Hospital), Changsha, Hunan, China

**Keywords:** PICU, children, accidental injuries PICU, accidental injuries, changing trend

## Abstract

**Objective:**

This study aimed to investigate the causes and clinical characteristics of 980 cases of accidental pediatric injuries admitted to the pediatric intensive care unit (PICU) to provide clinical evidence to support the prevention and reduction of severe accidental pediatric injuries.

**Methods:**

A total of 980 patients with accidental pediatric injuries admitted to the pediatric intensive care unit (PICU) of Hunan Children's Hospital from 2017 to 2023 were included in this study.

**Results:**

Between 2017 and 2023, 980 patients with accidental pediatric injuries were admitted to the PICU, comprising 588 boys and 392 girls (boy-to-girl ratio: 1.5:1). During the study period, a total of 16,151 children were admitted to the PICU, of whom 980 were admitted due to accidental injuries and 15,171 due to non-accidental injuries. There were no statistically significant differences in sex distribution between the accidental and non-accidental injury groups. Accidental pediatric injuries were most common among infants, toddlers, and preschool children, with the number of PICU admissions decreasing with increasing age. Across all years, infants and preschool children were the most affected. Traffic accidents and falls showed an increasing trend over time, whereas poisoning and drowning showed a decreasing trend. Differences in the composition of causes by year were statistically significant. The overall mortality rate among children with accidental injuries was 4.39%. The mortality rates by cause were as follows: traffic accidents (4.2%), falls (3.2%), foreign objects (7.1%), carbon monoxide poisoning (0.0%), food poisoning (9.7%), drug poisoning (1.0%), other types of poisoning (4.2%), burns and corrosive injuries (0.0%), drowning (13.0%), suffocation syndrome (23.1%), and other causes (4.3%). Prognostic differences between causes were statistically significant.

**Conclusion:**

Accidental pediatric injuries predominantly occur in boys, with infants and preschool children being the most affected. These injuries are more common in summer, with drug poisoning, traffic accidents, and falls being the main causes. Among the common causes, traffic accidents and falls showed an increasing trend, whereas poisoning and drowning showed a decreasing trend. Suffocation syndrome, drowning, and food poisoning were associated with high mortality rates.

## 1 Introduction

Accidental injuries refer to sudden and dangerous events in daily life that threaten an individual's health and life (1). These injuries are among the leading causes of death, hospitalization, and disability worldwide. For years, the issue of accidental pediatric injuries has been largely overlooked. Discussions on pediatric survival initiatives on the global agenda have seldom addressed this problem. Accidental pediatric injuries represent a critical public health issue that demands urgent attention.

Globally, more than 2,000 children and adolescents younger than 19 years die every day from preventable injuries. In China, accidental injuries are also a leading cause of death among children (2). Nowadays, data on accidental pediatric injuries requiring pediatric intensive care unit (PICU) care are limited. In this study, we collected data from the medical records of pediatric patients with accidental injuries who were admitted to the PICU of Hunan Children's Hospital from 2017 to 2023. The causes of injuries and clinical characteristics of patients with such injuries were analyzed to provide evidence-based data for preventing severe accidental pediatric injuries. The data were collected from one of the oldest tertiary-level specialized children's hospitals in China, which is the only one in the province. The PICU of Hunan Children's Hospital is also a nationally recognized clinical specialty unit. As such, the patients with severe accidental injuries admitted to this hospital are relatively representative. In this study, we analyzed the causes of accidental pediatric injuries and clinical characteristics of patients with such injuries over the past 7 years to provide evidence-based strategies for prevention and reduction.

## 2 Materials and methods

### 2.1 Data sources

Data were collected from pediatric patients admitted to the PICU of Hunan Children's Hospital due to accidental injuries between 1 January 2017 and 31 December 2023. Diagnoses were classified into the following categories based on the International Classification of Diseases, Tenth Revision (ICD-10): W00 (fall injuries), V01 (traffic injuries), T15 (injuries caused by foreign objects), T20 (burns/scalds and corrosive injuries), X40 (poisoning), P28 (suffocation syndrome), W65 (drowning), and T66 (other unspecified effects). A total of 980 patients were included in the study. Data collection included general information about the patients, such as their age, sex, time of admission, primary diagnosis, and prognosis.

### 2.2 Research methods

Statistical analysis was performed using SPSS 25.0. Categorical data were analyzed using the chi-square test, whereas qualitative data were expressed as frequencies and percentages. Comparisons of differences between groups for the categorical variables were conducted using the chi-square test, and Fisher's exact test was used when the conditions were not met. A *p*-value of <0.05 was considered statistically significant.

## 3 Results

### 3.1 Sex distribution of patients with accidental pediatric injuries

A total of 980 pediatric patients, including 588 boys and 392 girls, were admitted to the PICU for accidental injuries between 2017 and 2023. The distribution of visits to PICU of sex in different years from 2017 to 2023 is shown in Table 1. The boy-to-girl ratio for accidental injuries was 1.5:1. Across all years, the number of boys admitted to the PICU for accidental injuries was greater than that of girls, although the differences in sex composition were not statistically significant. During this period, 16,151 patients in total, including 980 with accidental injuries and 15,171 with non-accidental injuries, were admitted to the PICU. Sex distribution of the unexpected and expected groups between 2017 and 2023 is shown in Table 2. There were no statistically significant differences in sex distribution between the accidental and non-accidental injury groups.

**Table 1:** Distribution of visits to the PICU by sex in different years from 2017 to 2023.

Sex	2017	2018	2019	2020	2021	2022	2023
Boys	93	102	78	71	66	54	124
Girls	74	49	62	55	44	43	65
*χ* ^2^			9.885				

*p* = 0.130.

**Table 2:** Sex distribution of the unexpected and expected groups between 2017 and 2023.

Group	Boys	Girls	Total
Accident injury	588 (60%)	392 (40%)	980
Non-accidental injury	9,438 (62.2%)	5,733 (37.8%)	15,171
*χ*^2^ 1.911			
*p* = 0.167			

### 3.2 Age distribution of patients with accidental pediatric injuries

From 2017 to 2023, the age distribution of the 980 patients with accidental pediatric injuries admitted to the PICU was as follows:

• Under 3 years: 440 patients (44.9%)
• 3–5 years: 276 patients (28.2%)
• 6–8 years: 127 patients (13.0%)
• 9–11 years: 79 patients (8.1%)
• 12 years and older: 58 patients (5.9%)

Infants, toddlers, and preschool children were most commonly admitted for accidental injuries. The number of PICU admissions for accidental injuries tended to decrease with increasing age. Across all years, infants and preschool children were consistently the most affected. The differences in age distribution between years were statistically significant. The distribution of accidental pediatric injuries from 2017 to 2023 is shown in Table 3.

**Table 3:** Distribution of accidental pediatric injuries from 2017 to 2023.

Age (years)	2017	2018	2019	2020	2021	2022	2023	Total
<3	94	78	85	59	35	36	53	440
3–5	42	46	29	41	29	32	57	276
6–8	16	13	16	12	25	13	32	127
9–11	9	7	8	8	7	10	30	79
≧12	6	7	2	6	14	6	17	58
*χ* ^2^				41.732				
*p*				0.001				

### 3.3 Seasonal distribution of accidental pediatric injuries

From 2017 to 2023, 980 patients with accidental pediatric injuries were admitted most frequently during the summer. Differences in the seasonal distributions of accidental injuries across years were statistically significant. The distribution of accidental injuries in children admitted to the PICU from 2017 to 2023 is shown in Table 4.

**Table 4:** Distribution of accidental injuries in children admitted to the PICU from 2017 to 2023.

Year	Spring (March–May)	Summer (June–August)	Autumn (September–November)	Winter (December–February)
2017	34	50	50	33
2018	45	39	31	36
2019	28	44	38	30
2020	29	31	36	30
2021	28	26	28	28
2022	31	31	20	15
2023	56	69	17	47
Total	251	290	220	219
*χ* ^2^	41.732			
*p*	0.001			

### 3.4 Annual distribution of causes of accidental pediatric injuries

From 2017 to 2023, the causes of accidental injuries among PICU patients were ranked as follows:

• Poisoning: 391 patients
• Traffic accidents: 237 patients
• Falls: 154 patients
• Drowning: 100 patients
• Burns/scalds and corrosive injuries: 35 patients
• Suffocation syndrome: 26 patients
• Other unspecified effects: 23 patients
• Foreign objects: 14 patients

**Figure 1:**
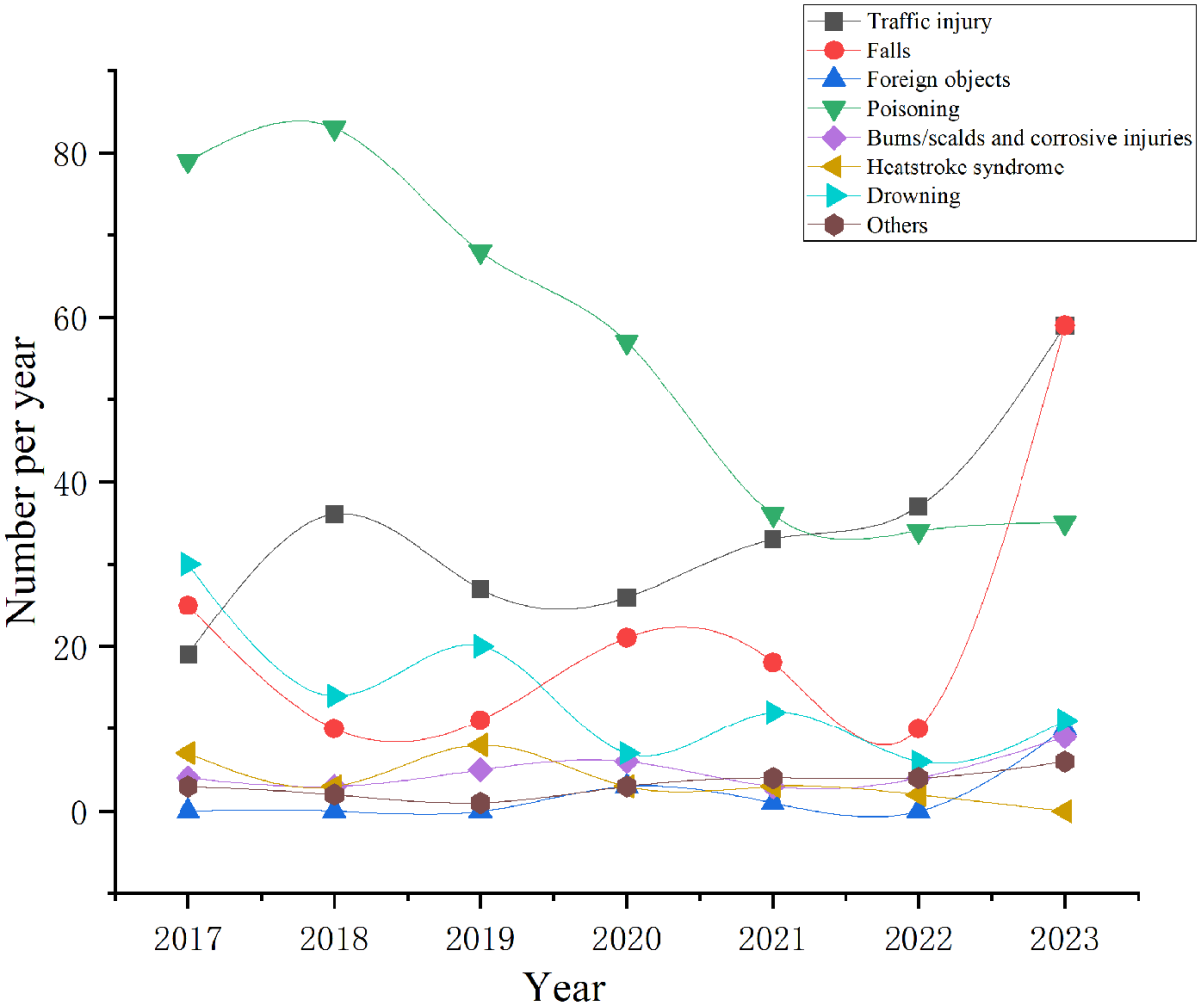
The changing trend of different causes of injury in children in PICU from 2017 to 2023.

Poisoning, traffic accidents, and falls were the primary causes of accidental pediatric injuries. In different years, poisoning remained the leading cause. Traffic accidents and falls showed an increasing trend, whereas poisoning, drowning, and suffocation syndrome demonstrated a decreasing trend. The differences in the composition of causes by year were statistically significant. The distribution of the causes of injuries in PICU patients from 2017 to 2023 is shown in Table 5. The changing trend of different causes of injury in children in PICU from 2017 to 2023 is shown in Figure 1, indicating an increasing trend of traffic accidents and falls and a decreasing trend of poisoning and drowning. The annual proportion of the causes of accidental injuries in 2017–2023 is shown in Figrure 2. The proportion of accidental injuries from 2017 to 2023 is shown in Figure 3.

**Table 5:** Distribution of the causes of injuries in PICU patients from 2017 to 2023.

Cause of disease	2017	2018	2019	2020	2021	2022	2023
Traffic injury	19 (11.4%)	36 (23.8%)	27 (19.3%)	26 (20.6%)	33 (30.0%)	37 (38.1%)	59 (31.2%)
Falls	25 (15.0%)	10 (6.6%)	11 (7.9%)	21 (16.7%)	18 (16.4%)	10 (10.3%)	59 (31.2%)
Foreign objects	0 (0.0%)	0 (0.0%)	0 (0.0%)	3 (2.4%)	1 (0.9%)	0 (0.0%)	10 (5.3%)
Poisoning	79 (47.3%)	83 (55.0%)	68 (48.6%)	57 (45.2%)	36 (32.7%)	34 (35.1%)	35 (18.5%)
Burns/scalds and corrosive injuries	4 (2.4%)	3 (2.0%)	5 (3.6%)	6 (4.8%)	3 (2.7%)	4 (4.1%)	9 (4.8%)
Heatstroke syndrome	7 (4.2%)	3 (2.0%)	8 (5.7%)	3 (2.4%)	3 (2.7%)	2 (2.1%)	0 (0.0%)
Drowning	30 (18.0%)	14 (9.3%)	20 (14.3%)	7 (5.6%)	12 (10.9%)	6 (6.2%)	11 (5.8%)
Others	3 (1.8%)	2 (1.3%)	1 (0.7%)	3 (2.4%)	4 (3.6%)	4 (4.1%)	6 (3.2%)
*χ* ^2^			177.954				
*p*			<0.001				

**Figure 2:**
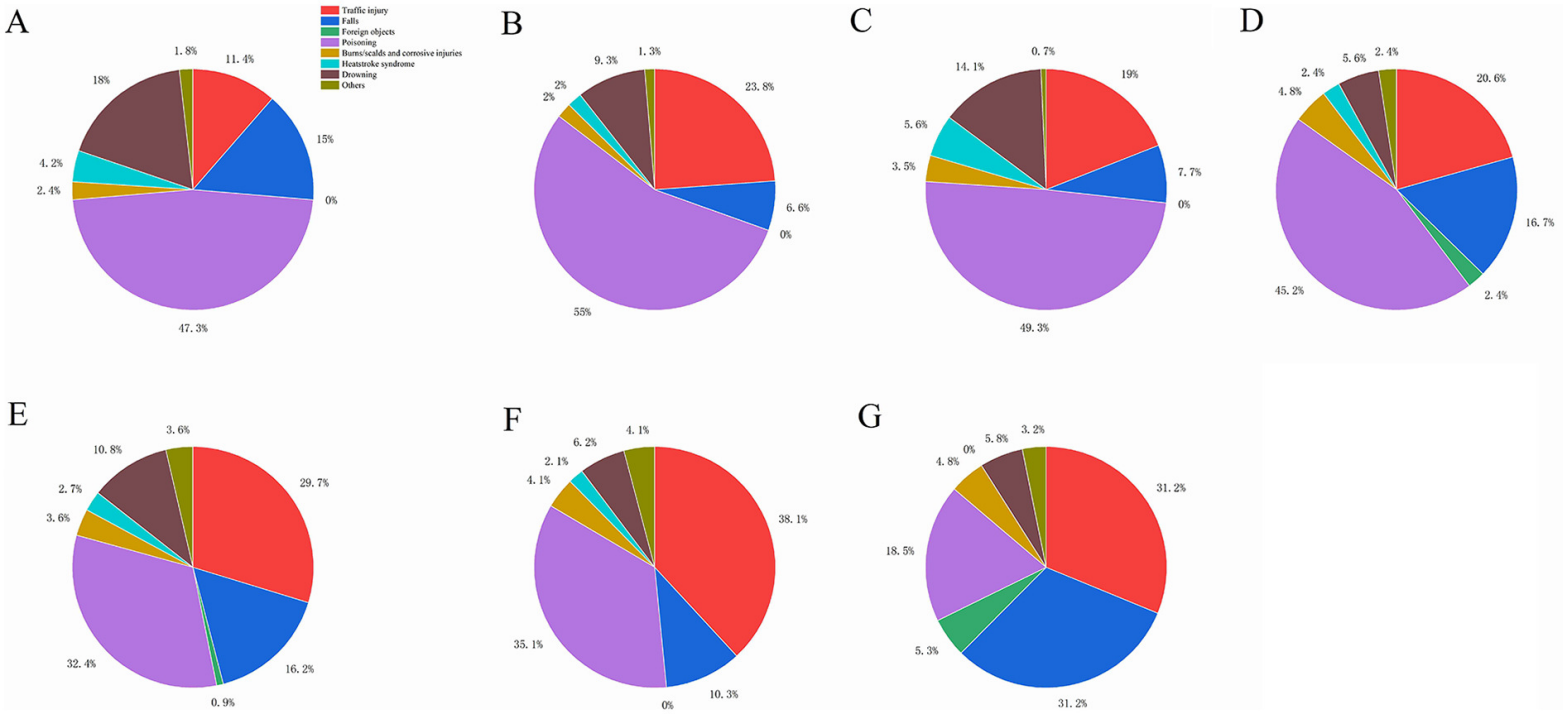
The annual proportion of the causes of accidental injuries in 2017–2023. **(A)** The annual proportion of the causes of accidental injuries in 2017. **(B)** The annual proportion of the causes of accidental injuries in 2018. **(C)** The annual proportion of the causes of accidental injuries in 2019. **(D)** The annual proportion of the causes of accidental injuries in 2020. **(E)** The annual proportion of the causes of accidental injuries in 2021. **(F)** The annual proportion of the causes of accidental injuries in 2022. **(G)** The annual proportion of the causes of accidental injuries in 2023.

**Figure 3:**
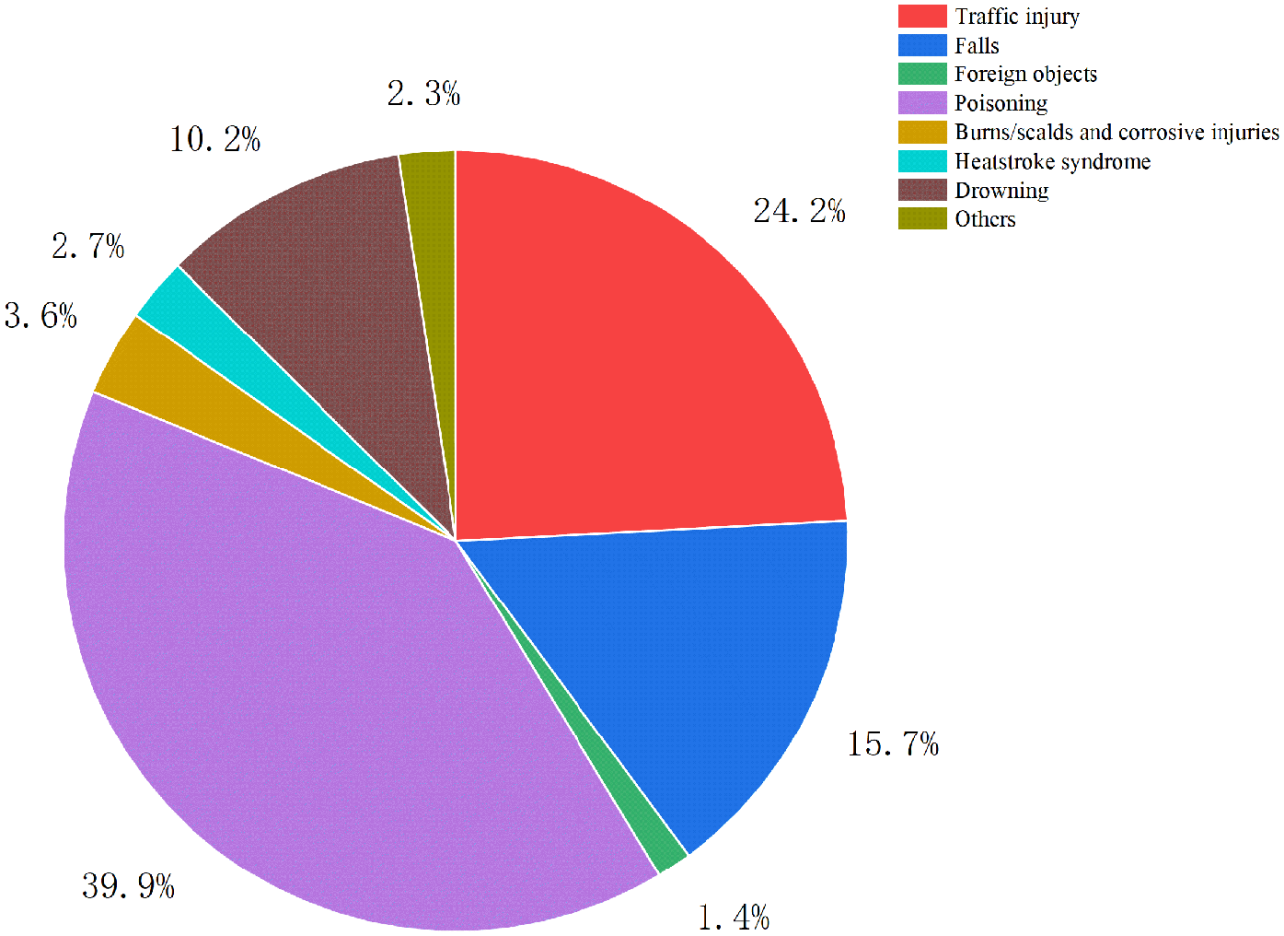
The portion of accidental injuries from 2017 to 2023.

### 3.5 Analysis of the general clinical data for different causes of accidental injuries in PICU patients

Among the 980 patients with accidental pediatric injuries, 588 were boys and 392 were girls. Across all types of injuries, the most common injuries for both sexes were caused by drug poisoning, traffic accidents, and falls. The incidence rates for all causes were higher among boys than among girls, but the difference in the sex composition was not statistically significant.

The age distribution was as follows: infancy and toddlerhood, preschool-age, and school-age. The proportions of accidental injuries varied across age groups. For infants and toddlers, the top three causes of accidental injuries were drug poisoning, drowning, and falls/traffic accidents. For preschool-aged and school-aged children, the top three causes of accidental injuries were traffic accidents, drug poisoning, and falls. For adolescents, the top three causes of accidental injuries were drug poisoning, falls, and traffic accidents. The differences in age composition for various causes were statistically significant.

The seasonal distribution rankings are as follows: summer, autumn, spring, and winter. Across all seasons, the primary causes of accidental injuries were drug poisoning, traffic accidents, and falls. The differences in seasonal composition for various causes were statistically significant. General clinical data of children with different types of accidental injuries are shown in Table 6.

**Table 6:** General clinical data of children with different types of accidental injuries.

General clinical data	Type of accidental injury												*χ* ^2^	*p*
	Traffic injury	Falls	Foreign objects	Poisoning				Burns/scalds and corrosive injuries	Drowning	Heatstroke syndrome	Others	Total		
				Carbon monoxide	Food	Medicine	Other poisoning							
Sex														
Boys	145 (61.2%)	108 (70.1%)	11 (78.6%)	18 (52.9%)	20 (64.5%)	157 (52.0%)	15 (62.5%)	20 (57.1%)	66 (66.0%)	15 (57.7%)	13 (56.5%)	588 (60%)	19.639	0.033
Girls	92 (38.8%)	46 (29.9%)	3 (21.4%)	16 (47.1%)	11 (35.5%)	145 (48.0%)	9 (37.5%)	15 (42.9%)	34 (34.0%)	11 (42.3%)	10 (43.5%)	392 (40%)		
Age														
Infant	53 (22.4%)	53 (34.4%)	12 (85.7%)	16 (47.1%)	9 (29.0%)	158 (52.3%)	13 (54.2%)	26 (74.3%)	65 (65.0%)	26 (100.0%)	9 (39.1%)	440 (44.9%)	185.600	<0.001
Preschool-age	98 (41.4%)	47 (30.5%)	1 (7.1%)	4 (11.8%)	9 (29.0%)	81 (26.8%)	5 (20.8%)	5 (14.3%)	20 (20.0%)	0 (0.0%)	6 (26.1%)	276 (28.2%)		
School-age	78 (32.9%)	43 (27.9%)	1 (7.1%)	14 (41.2%)	9 (29.0%)	33 (10.9%)	4 (16.7%)	4 (11.4%)	13 (13.0%)	0 (0.0%)	7 (30.4%)	206 (21.0%)		
Adolescent	8 (3.4%)	11 (7.1%)	0 (0.0%)	0 (0.0%)	4 (12.9%)	30 (9.9%)	2 (8.3%)	0 (0.0%)	2 (2.0%)	0 (0.0%)	1 (4.3%)	58 (5.9%)		
Season														
Spring	57 (24.1%)	44 (28.6)	3 (21.4%)	5 (14.7%)	6 (19.4%)	84 (27.8%)	7 (29.2%)	6 (17.1%)	27 (27.0%)	4 (15.4%)	8 (34.8%)	251 (25.6%)		
Summer	85 (35.9%)	46 (29.9)	1 (7.1%)	2 (5.9%)	20 (64.5%)	79 (26.2%)	3 (12.5%)	16 (45.7%)	33 (33.0%)	1 (3.8%)	4 (17.4%)	290 (29.6%)	122.046	<0.001
Autumn	46 (19.4%)	37 (24.0)	4 (28.6%)	6 (17.6%)	4 (12.9%)	78 (25.8%)	9 (37.5%)	6 (17.1%)	20 (20.0%)	2 (7.7%)	8 (34.8%)	220 (22.4%)		
Winter	49 (20.7%)	27 (17.5%)	6 (42.9%)	21 (61.8%)	1 (3.2%)	61 (20.2%)	5 (20.8%)	7 (20.0%)	20 (20.0%)	19 (73.1%)	3 (13.0%)	219 (22.3%)		
Prognosis														
Cure	21 (8.9%)	13 (8.4%)	5 (35.7%)	0 (0.0%)	3 (9.7%)	44 (14.6%)	2 (8.3%)	4 (11.4%)	10 (10.0%)	0 (0.0%)	1 (4.3%)	103 (10.5%)		
Improve	202 (85.2%)	127 (82.5%)	7 (50.0%)	31 (91.2%)	23 (74.2%)	250 (82.8%)	21 (87.5%)	29 (82.9%)	74 (74.0%)	20 (76.9%)	19 (82.6%)	803 (81.9%)	93.662	<0.001
Not more	4 (1.7%)	9 (5.8%)	1 (7.1%)	3 (8.8%)	2 (6.5%)	5 (1.7%)	0 (0.0%)	2 (5.7%)	3 (3.0%)	0 (0.0%)	2 (8.7%)	31 (3.2%)		
Death	10 (4.2%)	5 (3.2%)	1 (7.1%)	0 (0.0%)	3 (9.7%)	3 (1.0%)	1 (4.2%)	0 (0.0%)	13 (13.0%)	6 (23.1%)	1 (4.3%)	43 (4.4%)		
Total	237	154	14	34	31	302	24	35	100	26	23	980		

The prognosis outcomes were as follows: full recovery (10.5%), improvement (81.9%), no improvement (3.2%), and death (4.4%). The differences in prognosis composition among the causes were statistically significant.

The direct cause of death for nine children of car accident injuries and five children of elevated fall injuries in children was cerebral hemorrhage. The direct cause of death for 13 children who drowned was drowning. The direct cause of death in six children with heatstroke syndrome was multiple organ dysfunction. Among the seven children who died from poisoning, the causes include one case of arsenic poisoning, one case of botulism, two cases of toxin poisoning, one case of paraquat poisoning, one case of amoxicillin poisoning, and one case of chlorpromazine poisoning. The direct cause of death in another case of oxygen tank injury was cerebral hemorrhage.

The mortality rates for specific causes were as follows:

• Traffic accidents: 4.2%
• Falls: 3.2%
• Foreign objects: 7.1%
• Carbon monoxide poisoning: 0.0%
• Food poisoning: 9.7%
• Drug poisoning: 1.0%
• Other types of poisoning: 4.2%
• Burns/scalds and corrosive injuries: 0.0%
• Drowning: 13.0%
• Suffocation syndrome: 23.1%
• Other causes: 4.3%

## 4 Discussion

### 4.1 Overview of accidental pediatric injuries

This retrospective study was conducted at Hunan Children's Hospital from 2017 to 2023 and focused on patients with accidental pediatric injuries who were admitted to the PICU. Over the past few decades, while significant progress has been made in healthcare, resulting in marked reductions in the number of deaths from infectious diseases and cancer, deaths among children and adolescents related to injuries have steadily increased (3). Accidental injuries pose a severe threat to the safety and lives of children worldwide. In 2015, the WHO European Region reported 18,328 pediatric deaths due to injuries, with 88.5% resulting from accidental causes (4).

Children are highly susceptible to accidental injuries because their ability to recognize and respond to risks is relatively weak, increasing their vulnerability to potential dangers. Moreover, severe accidental injuries in children result in high rates of disability and mortality, significantly affecting their quality of life and long-term development.

### 4.2 Sex and age distribution of patients with accidental pediatric injuries

This study revealed that, from 2017 to 2023, 588 boys and 392 girls were admitted to the PICU for accidental injuries, with a boy-to-girl ratio of 1.5:1. Across all years, the number of boys exceeded that of girls. For different causes of accidental injuries, boys were consistently more affected than girls were. This may be due to several reasons. (1) Studies have suggested that boys are generally more active, curious, and inclined to explore unknown environments and try new things, leading to a greater risk of accidental injuries (5). (2) Boys and girls exhibit different motivation levels and behavioral patterns (6). Boys are more likely to engage in risky activities, increasing their likelihood of experiencing accidental injuries.

The age distribution revealed a decreasing trend in the incidence of PICU admissions for accidental injuries with increasing age. Infants and preschool children accounted for the majority of accidental injury cases. This is primarily because younger children have weaker motor balance, risk responses, and emergency perception abilities than older children do, making them more prone to losing control during activities, resulting in falls or collisions.

With age, children develop improved cognitive abilities and a stronger sense of self-protection, enabling them to better recognize and avoid potential dangers, thus reducing their risk of accidental injuries. Other factors contributing to higher rates of accidental injuries in younger children include incomplete development, heightened curiosity, and a higher center of gravity during movement (7).

Preschool-aged children represent a high-risk group, partly because of increased independence, more challenging behavior, and reduced adult supervision (8), resulting in higher injury rates. The leading causes of injuries by age group were as follows:

• Infants: drug poisoning, drowning, falls, and traffic accidents
• Preschool-aged and school-aged children: traffic accidents, drug poisoning, and falls
• Adolescents: drug poisoning, falls, and traffic accidents

While drug poisoning, traffic accidents, and falls were common across all age groups, the proportions varied significantly. Different developmental stages influence children's ability to recognize and respond to risks. For example, cognitive levels increase with age, enabling better recognition of hazardous factors.

The most common age-specific injury was suffocation syndrome, which was almost exclusively observed in infants under 1 year of age. This syndrome largely occurs due to improper caregiving practices. Many parents overdress their children or cover them excessively with blankets due to fear of cold, even obstructing their child's nose and mouth. This creates a high-temperature, low-oxygen environment.

Infants lack a fully developed thermoregulatory center, preventing effective body temperature regulation. Additionally, their underdeveloped language skills hinder their ability to cry for help, increasing the likelihood of accidental injuries.

### 4.3 Annual trends in PICU admissions for accidental pediatric injuries

The study results indicate that the number of accidental pediatric injuries admitted to the PICU tended to decrease from 2017 to 2022 but significantly increased in 2023.

On the one hand, studies have suggested that the spectrum of diseases among PICU patients changed notably during the 2 years of the coronavirus disease 2019 (COVID-19) pandemic, with a significant decrease in the number of critically ill pediatric patients (9). This could be attributed to effective epidemic prevention measures that reduce the incidence and progression of certain diseases. Parents working from home during this period were more attentive to their children, thereby reducing the risk of accidental injuries. In addition, improvements in online medical consultations and the dissemination of knowledge about preventing and treating accidental pediatric injuries may have contributed to reduced demand for hospital visits and fewer critical conditions resulting from accidental injuries (10, 11).

On the other hand, the Chinese government has increasingly prioritized children's well-being, launching policies and public education campaigns to prevent accidental injuries. For example, initiatives such as the “National Action Plan for Disability Prevention (2016–2020)” and the “2015 Guidelines on Health Literacy for Chinese Citizens: Basic Knowledge and Skills,” as well as a series of technical guidelines for injury intervention, have effectively reduced the incidence of accidental injuries and, consequently, PICU admissions.

However, related studies suggest that many children do not receive timely treatment for injuries, even in fatal cases. This indicates that some critically injured children may not have received timely prehospital treatment due to various factors (12).

The sharp increase in the number of PICU admissions for accidental injuries in 2023 can be attributed to the following factors:

In December 2022, the optimization of China's pandemic control measures and the gradual return to normal in work and daily life led to weakened safety awareness among parents. Parents may have focused more on restoring their lives and work and paid relatively less attention to childcare and safety education.
As normal life resumed, outdoor activities for both parents and children increased, along with the number of vehicles on the roads, leading to an increase in accidental injuries among children.
Research indicates that the increase in post-pandemic accidental injuries may also be related to the psychological impact of pandemic prevention measures on children and adolescents. Prolonged home isolation and social restrictions may have caused anxiety, depression, and other psychological issues among children and adolescents. These issues could negatively affect child behavior and judgment, thereby increasing the risk of accidental injuries.

### 4.4 General clinical data on accidental pediatric injuries by cause

The study findings indicated the following distribution of causes among patients with accidental pediatric injuries: poisoning (393 patients), traffic accidents (237 patients), falls (154 patients), drowning (100 patients), burns/scalds and corrosive injuries (35 patients), suffocation syndrome (26 patients), other unspecified effects (23 patients), and foreign objects (14 patients). Poisoning, traffic accidents, and falls were the most common causes, although the incidence rates reported in this study differ from those reported in most existing studies (13).

Among the 393 poisoning cases treated in the PICU of Hunan Children's Hospital, 34 were due to carbon monoxide poisoning, 302 were due to drug poisoning, 33 were due to food poisoning, and 24 were due to other types of poisoning. Poisoning accounted for 40% of all accidental injuries, with the following incidence rates: carbon monoxide poisoning (3.5%), drug poisoning (30.8%), and food poisoning (3.2%). The mortality rates for these causes were as follows: carbon monoxide poisoning (0.0%), food poisoning (9.7%), and drug poisoning (1.0%).

Common causes of drug poisoning include rodenticides and medications used for mental health disorders and chronic illnesses. According to Arnulf Soleng, rodenticide poisoning accounts for 65% of poisoning cases across all age groups. Owing to agricultural practices and the demand for rodent control in the Hunan region, rodenticides are widely used, resulting in a high rate of accidental poisoning. Children are particularly prone to accidental poisoning due to improper storage of medicines and household substances.

Children's natural curiosity and habit of exploring the world through touch and taste increase their risk of mistaking candy-like or food-packaged medicines as consumables. Additionally, the increasing use of prescription and over-the-counter drugs, including painkillers and psychotropic medications, has been identified as a potential driver of poisoning cases (14).

Despite the high proportion of accidental injuries, cases of poisoning requiring PICU care have shown a decreasing trend. The reasons include increased awareness of poisoning risks due to public education campaigns, stricter government regulation of pesticides, and measures such as warning labels on hazardous substances in public spaces. Parents' increased safety consciousness and improved storage of medicines and household chemicals have also reduced children's exposure to poisonous substances. Advances in medical care, particularly in early recognition and timely treatment, have enabled many patients with poisoning to stabilize after emergency interventions without requiring further intensive care.

Drowning is a regionally influenced cause of accidental injuries. In 2017, reports indicated that China, India, Pakistan, and Bangladesh accounted for 51.2% of drowning deaths worldwide (15), with China ranking fourth in drowning mortality among G20 nations (16). Children who experience drowning and are treated in the PICU predominantly include infants and toddlers, who are at increased risk owing to their wide activity range and tendency to approach water sources.

Unprotected water areas, such as ponds, rivers, and lakes, lack essential safety measures such as fences and warning signs, increasing the risk of accidental drowning among children. The underdeveloped physical coordination and self-protection abilities of infants and toddlers, coupled with inadequate supervision, further contribute to drowning incidents. Drowning has a high mortality rate, primarily because timely and effective rescue is critical. A delayed emergency response often results in secondary complications such as hypoxic brain injury or acute traumatic coagulopathy (ATC), contributing to increased mortality rates. However, drowning cases have shown a decreasing trend, possibly due to increased drowning prevention awareness and widespread training in emergency rescue measures.

Traffic accidents are the leading cause of death for children aged 0–14 years, accounting for 34% of child injury deaths annually (17). Motor vehicle collisions account for nearly 50% of fatal injuries among children, with higher rates of pedestrian injuries in children than in adults (18). The number of traffic accident cases treated in the PICU increased annually from 2017 to 2023.

The contributing factors include the increasing number of vehicles, inadequate road safety measures, and children's engagement in risky behaviors such as crossing streets unsafely or playing on roads. Research suggests that motor vehicle-related collisions are more likely to result in severe injuries requiring PICU admission (19).

Similarly, falls have shown an increasing trend, reflecting children's expanding activity range into high-rise spaces due to modern urbanization. The absence of proper safety measures, combined with children's inclination to climb and limited understanding of risks, increases the likelihood of falls. While falls can occur across all age groups, younger children are particularly vulnerable because of their inability to control their bodies effectively during falls, increasing impact force.

Foreign object-related injuries are relatively rare in the PICU, likely because such cases are often resolved in outpatient or emergency settings. Commonly affected areas include the respiratory, digestive, and urinary tracts, where objects are safely removed without requiring intensive care.

The incidence of burns and scalds has also decreased, which may be attributed to the reduced use of old-fashioned hot water bottles, bans on fireworks, increased public education, and improved product safety standards.

From 2017 to 2023, the overall PICU mortality rate for accidental injuries was approximately 4.4%. Poisoning, traffic accidents, and falls had high incidence rates, whereas suffocation syndrome, drowning, and food poisoning had the highest mortality rates. These findings differ from the national mortality data reported in the “China Cause of Death Surveillance Dataset,” potentially reflecting differences in treatment timeliness and quality.

### 4.5 Seasonal characteristics of accidental pediatric injuries

This study revealed that accidental pediatric injuries were most common in summer. Studies suggest that the arrival of summer brings more sunlight, increased outdoor time, and more opportunities for recreation and play, which also increases the risk of accidental injuries.

Certain seasonal injuries, such as suffocation syndrome, were concentrated in the winter and spring, and carbon monoxide poisoning also primarily occurred during these seasons. Additionally, studies indicate that drowning is related to environmental temperatures, with increased drowning rates with increasing temperature (20). However, pediatric drowning cases were relatively evenly distributed across all seasons, with cases reported throughout the year.

Research shows that drowning locations vary by age group:

• Ages 1–4: primarily indoors (bathtubs, water basins, and buckets)
• Ages 5–9: canals, ponds, and reservoirs
• Ages 10 and above: ponds, lakes, and rivers

The poor coordination and motor skills of young children, combined with a lack of awareness of risks, often cause them to fall into areas of water, endangering their lives. These incidents can occur in any season, especially when supervision is insufficient (21).

In summary, patients with accidental pediatric injuries treated in the PICU were analyzed in this study. Poisoning, traffic accidents, and falls were the leading causes of accidental pediatric injuries, with incidence rates varying by sex, age, and season. Accidental injuries remain a leading cause of death among children. The “Children and Safety” section was included in China's “Outline for Children's Development (2021–2030)” to address new challenges in child safety. This initiative emphasizes reducing the incidence of accidental pediatric injuries and lowering mortality rates caused by such injuries. A thorough understanding of the causes and clinical characteristics of accidental pediatric injuries is crucial for developing targeted prevention measures to reduce their occurrence and related deaths.

## 5 Study limitations

This is a single-center and retrospective study. Thus, further multicenter studies conducted with larger sample sizes are needed.

## 6 Conclusion

Accidental pediatric injuries were predominantly observed in boys and were most common among infants and preschool children. Summer was the peak season for these injuries, and the leading causes were drug poisoning, traffic accidents, and falls. Among the common causes, traffic accidents and falls showed an increasing trend, whereas poisoning and drowning showed a decreasing trend. Children who experienced suffocation syndrome, drowning, and food poisoning had the highest mortality rates.

## Data Availability

The original contributions presented in the study are included in the article/Supplementary Material, further inquiries can be directed to the corresponding author.

## References

[1] Yin X Li D Zhu K Liang X Peng S Tan A Comparison of intentional and unintentional injuries among Chinese children and adolescents. J Epidemiol. (2020) 30(12):529–36. 10.2188/jea.JE20190152 31708510 PMC7661334

[2] Wang Z Chen H Yu T Liu S Hu M. Status of injuries as a public health burden among children and adolescents in China: a systematic review and meta-analysis. Medicine. (2019) 98(45):e17671. 10.1097/MD.0000000000017671 31702619 PMC6855559

[3] Dorney K Dodington JM Rees CA Farrell CA Hanson HR Lyons TW Preventing injuries must be a priority to prevent disease in the twenty-first century. Pediatr Res. (2020) 87(2):282–92. 10.1038/s41390-019-0549-7 31466080

[4] Sethi D Aldridge E Rakovac I Makhija A. Worsening inequalities in child injury deaths in the WHO European region. Int J Environ Res Public Health. (2017) 14(10):1128. 10.3390/ijerph14101128 28954422 PMC5664629

[5] Yu X Wang Y He C Kang L Miao L Wu Y The trend of unintentional injury-related mortality among children aged under-five years in China, 2010–2020: a retrospective analysis from a national surveillance system. BMC Public Health. (2023) 23(1):673. 10.1186/s12889-023-15546-6 37041562 PMC10088152

[6] Gong H Lu G Ma J Zheng J Hu F Liu J Causes and characteristics of children unintentional injuries in emergency department and its implications for prevention. Front Public Health. (2021) 9:669125. 10.3389/fpubh.2021.669125 34422741 PMC8374066

[7] Wang Z Hu Y Peng F. Long-term trends in unintentional fall mortality in China: a population-based age-period-cohort study. Front Public Health. (2021) 9:749295. 10.3389/fpubh.2021.749295 35024364 PMC8744467

[8] Flavin MP Dostaler SM Simpson K Brison RJ Pickett W. Stages of development and injury patterns in the early years: a population-based analysis. BMC Public Health. (2006) 6:187. 10.1186/1471-2458-6-187 16848890 PMC1569842

[9] Xin MY Wu JF Wang XS Han L. Changes in the disease spectrum in the pediatric intensive care units within 2 years before and after the outbreak of coronavirus disease 2019. Zhongguo Dang Dai Er Ke Za Zhi. (2022) 24(10):1098–103. (in Chinese). 10.7499/j.issn.1008-8830.2205074 36305109 PMC9627991

[10] Li Y Yan X Song X. Provision of paid web-based medical consultation in China: cross-sectional analysis of data from a medical consultation website. J Med Internet Res. (2019) 21(6):e12126. 10.2196/12126 31162129 PMC6746088

[11] Liu WY Tung TH Zhou Y Gu DT Chen HY. The relationship between knowledge, attitude, practice, and fall prevention for childhood in Shanghai, China. Front Public Health. (2022) 10:848122. 10.3389/fpubh.2022.848122 35359797 PMC8963735

[12] Zhou X Xie Z He J Lin H Xiao J Wang H Unintentional injury deaths among children under five in hunan province, China, 2015–2020. Sci Rep. (2023) 13(1):5530. 10.1038/s41598-023-32401-1 37016022 PMC10073091

[13] Li L Scherpbier R Wu J Zhu X Zhang W Zhang L Legislation coverage for child injury prevention in China. Bull World Health Organ. (2015) 93(3):169–75. 10.2471/BLT.14.139998 25838612 PMC4371490

[14] Li H Dodd-Butera T Beaman ML Pritty MB Heitritter TE Clark RF. Trends in childhood poison exposures and fatalities: a retrospective secondary data analysis of the 2009–2019 U.S. National Poison Data System Annual Reports. Pediatr Rep. (2021) 13(4):613–23. 10.3390/pediatric13040073 34842797 PMC8628925

[15] Franklin RC Peden AE Hamilton EB Bisignano C Castle CD Dingels ZV The burden of unintentional drowning: global, regional and national estimates of mortality from the global burden of disease 2017 study. Inj Prev. (2020) 26(1):i166. 10.1136/injuryprev-2019-043484corr1. *Inj Prev*. (2020) **26**(Supp 1):i83–i95. doi: 10.1136/injuryprev-2019-043484. 32079663 PMC7571364

[16] Haagsma JA Graetz N Bolliger I Naghavi M Higashi H Mullany EC The global burden of injury: incidence, mortality, disability-adjusted life years and time trends from the global burden of disease study 2013. Inj Prev. (2016) 22(1):3–18. 10.1136/injuryprev-2015-041616 26635210 PMC4752630

[17] Armour-Marshall J Wolfe I Richardson E Karanikolos M McKee M. Childhood deaths from injuries: trends and inequalities in Europe. Eur J Public Health. (2012) 22(1):61–5. 10.1093/eurpub/ckr004 21310718

[18] Bao Y Ye J Hu L Guan L Gao C Tan L. Epidemiological analysis of a 10-year retrospective study of pediatric trauma in intensive care. Sci Rep. (2024) 14(1):21058. 10.1038/s41598-024-72161-0 39256597 PMC11387635

[19] Chong S Poulos R Olivier J Watson WL Grzebieta R. Relative injury severity among vulnerable non-motorised road users: comparative analysis of injury arising from bicycle-motor vehicle and bicycle-pedestrian collisions. Accid Anal Prev. (2010) 42(1):290–6. 10.1016/j.aap.2009.08.006 19887170

[20] Huang Z Li Z Hu J Zhu S Gong W Zhou C The association of heatwave with drowning mortality in five provinces of China. Sci Total Environ. (2023) 903:166321. 10.1016/j.scitotenv.2023.166321 37586513

[21] Li L Zhang ZQ Zheng CZ Shi Y. Expert consensus on the prevention and treatment of drowning in children. Chinese Journal of Contemporary Pediatrics. (2021) 23(1):12–7. 10.7499/j.issn.1008-8830.2008005 33476531 PMC7818148

